# K-Module Algorithm: An Additional Step to Improve the Clustering Results of WGCNA Co-Expression Networks

**DOI:** 10.3390/genes12010087

**Published:** 2021-01-12

**Authors:** Jie Hou, Xiufen Ye, Chuanlong Li, Yixing Wang

**Affiliations:** College of Intelligent Systems Science and Engineering, Harbin Engineering University, Harbin 150001, China; houjie@hrbeu.edu.cn (J.H.); lcl@hrbeu.edu.cn (C.L.); wangyixing@lyu.edu.cn (Y.W.)

**Keywords:** gene co-expression networks, distance correlation, connectivity, enrichment analysis

## Abstract

Among biological networks, co-expression networks have been widely studied. One of the most commonly used pipelines for the construction of co-expression networks is weighted gene co-expression network analysis (WGCNA), which can identify highly co-expressed clusters of genes (modules). WGCNA identifies gene modules using hierarchical clustering. The major drawback of hierarchical clustering is that once two objects are clustered together, it cannot be reversed; thus, re-adjustment of the unbefitting decision is impossible. In this paper, we calculate the similarity matrix with the distance correlation for WGCNA to construct a gene co-expression network, and present a new approach called the k-module algorithm to improve the WGCNA clustering results. This method can assign all genes to the module with the highest mean connectivity with these genes. This algorithm re-adjusts the results of hierarchical clustering while retaining the advantages of the dynamic tree cut method. The validity of the algorithm is verified using six datasets from microarray and RNA-seq data. The k-module algorithm has fewer iterations, which leads to lower complexity. We verify that the gene modules obtained by the k-module algorithm have high enrichment scores and strong stability. Our method improves upon hierarchical clustering, and can be applied to general clustering algorithms based on the similarity matrix, not limited to gene co-expression network analysis.

## 1. Introduction

Gene co-expression networks are increasingly used to explore the system-level functionality of genes [1]. They have been widely studied and used for predicting new gene functions, discovering new disease biomarkers, and detecting genetic variants in cancers [2,3]. Network construction is conceptually straightforward: nodes represent genes and are connected if the corresponding genes are significantly co-expressed across samples [1,4].

The most commonly used pipelines for co-expression networks construction is weighted gene co-expression network analysis (WGCNA) [5]. WGCNA can be used for finding clusters (modules) of highly correlated genes, for summarizing such clusters using the module eigengene or intramodular hub gene, for relating modules to one another and to external sample traits (using eigengene network methodology), and for calculating module membership measures [6]. WGCNA identifies gene modules using unsupervised clustering, and the default method is hierarchical clustering [1]. Hierarchical clustering is a common method used to determine clusters of similar data points in multidimensional spaces [7]. It can be mainly categorized into agglomerative and divisive procedures. Agglomerative clustering uses a bottom-up approach, wherein each data point starts in its own cluster. These clusters are then joined by taking the two most similar clusters together and merging them. This merger continues until all the samples are clustered into one group. Consequently, a tree-like structure, known as a dendrogram, is produced. If the number of clusters is provided, the process of amalgamation of clusters can be terminated when the desired number of clusters is obtained [8,9]. WGCNA identifies gene modules using hierarchical clustering. Branches of the hierarchical clustering dendrogram correspond to modules, which can be identified using the dynamic tree cut method [10]. The dynamic tree cut method succeeds at identifying branches that could not have been identified using the static cut method. Prior studies provide indirect evidence that the resulting clusters are biologically meaningful [11].

Researchers have conducted considerable work to improve the standard of WGCNA. Greenfest-Allen et al. [12] improved the robustness of the whole-transcriptome gene correlation network clustering by pruning poorly fitting genes from estimated modules and then rerunning WGCNA to refine gene clusters. Dai et al. [13] introduced a modified method of WGCNA, known as the combination of signed and unsigned WGCNA (csuWGCNA). Toubiana et al. [14] developed a stochastic optimization algorithm, known as the genetic algorithm, optimizing the trait-to-gene module relationship by gradually increasing the correlation between the trait and a subset of genes of the gene module. However, hierarchical clustering was used in all the above algorithms, and its disadvantages were ignored. Botía et al. [15] proposed k-means clustering as an additional processing step to the standard WGCNA. Their definition of k-means used eigengenes as centroids, and these specific types of networks separate upregulated genes from downregulated genes in different modules. Using the eigengenes as the centroids in general networks (networks that are not separated into up- and downregulated genes) may lead to some problems. In practice, genes in a module may not always be highly correlated (in absolute terms). In such cases, using the eigengene (defined as the first principal component of the module’s expression matrix) is likely inappropriate, as it will not be representative of the module.

Due to the characteristics of the algorithm, hierarchical clustering may assigns a gene to a module just because the module contains one gene that has the highest correlation with this gene. Since the relationship between genes is networked, more genes that have higher correlations should be considered. The hierarchical clustering does not have a global goal; therefore, we wanted to reverse and re-adjust the decisions found in hierarchical clustering results, and simultaneously retain the advantages of the dynamic tree cut method. In other words, our objective was to keep the number of modules constant while a small number of decisions are re-adjusted. Our goal was to globally find cluster (module) for each gene in which the gene has a high mean connectivity to the other genes. In this paper, we calculate the similarity matrix with the distance correlation for WGCNA to construct a gene co-expression network. Then, we present an additional post-processing step for the hierarchical clustering of WGCNA, called the k-module algorithm. We verified that this k-module algorithm, together with WGCNA, performs better than the standard WGCNA in some aspects, such as enrichment analysis and module stability analysis.

## 2. Methods

### 2.1. WGCNA and k-Module Algorithm

**Figure 1:**
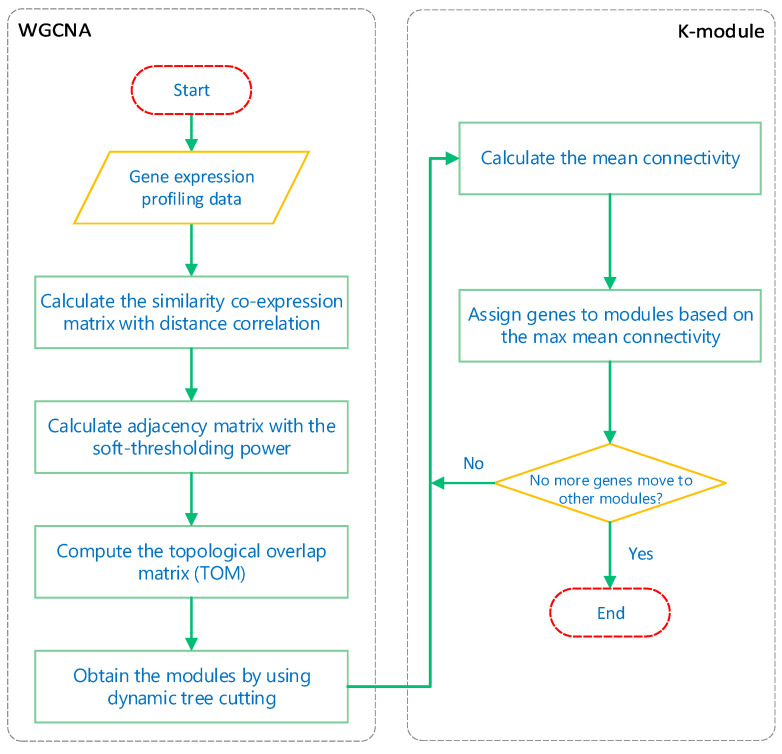
Weighted gene co-expression network analysis (WGCNA) and k-module algorithm flow chart.

In this study, we sought to obtain modules with highly correlated member genes, so we designed the k-module algorithm to improve the clustering results of WGCNA. The flow chart of our algorithm is shown in Figure 1. The flow chart is divided into two stages: WGCNA and the k-module algorithm.

In the first stage, we mainly demonstrate the process of module-obtaining in WGCNA. Firstly, the similarity co-expression matrix $s_{ij}$ is calculated for all genes, where $s_{ij}=\left|\mathrm{cor}\left(x_{i},x_{j}\right)\right|$ is the absolute value of the correlation coefficient between the gene expression profiles of nodes *i* and *j*. In this paper, the correlation coefficients are calculated by distance correlation. Since distance correlation coefficients are always positive, they define an unsigned network in which positive and negative correlations are treated equally. Then, the similarity co-expression matrix is transformed into an adjacency matrix $a_{ij}$ by setting $a_{ij}=s_{ij}^{\beta }$, with β as the soft-thresholding power. The soft-thresholding power is chosen based on the criteria of approximating the scale-free topology (SFT) of the network [1]. Next, a topological overlap matrix is computed from the adjacency matrix. Finally, hierarchical clustering is used to produce a tree (dendrogram) from the dissimilarity topological overlap matrix. Using dynamic tree cutting, different numbers of clusters (modules) are obtained.

The second stage is the k-module algorithm proposed in this paper. The input of the k-module algorithm is the gene modules obtained by WGCNA. The main purpose of the algorithm is to calculate the mean connectivity of the genes in each module, and assign the genes to the modules to which they most highly connect. Here, the mean connectivity of the gene $g_{i}$ to the *m*th module is defined as:

(1) $$\frac{1}{n_{m}}\sum_{g_{j}\in S_{m}}a_{ij}$$

where $n_{m}$ is the gene number of the *m*th module and $g_{j}$ belongs to $S_{m}$, the set of all genes in the *m*th module. For each gene $g_{i}$, we set the module label of $g_{i}$ to:

(2) $$\arg\max_{m}\frac{1}{n_{m}}\sum_{g_{j}\in S_{m}}a_{ij}$$

Then, we recalculate the mean connectivity of the genes in each module and repeat the previous steps until none of the cluster assignments change or the iterations reach the set maximum number. The R code of the k-module algorithm can be found in Supplementary Material 1.

### 2.2. Distance Correlation

Distance correlation was proposed in 2007 by Szekely et al. It has a perfect theoretical system and works for both linear and nonlinear dependencies [16,17]. The Pearson correlation coefficient, as a linear dependence measure, is the most common default measure among co-expression network tools. However, the true relationships observed in a biological system are complex [18], involving not only linear dependence but also some nonlinear dependencies. Therefore, in this study, we chose distance correlation to measure the correlations between the gene expression profiles. The distance correlation was calculated using the energy package in R (https://CRAN.R-project.org/package=energy).

### 2.3. An Algorithm Using Eigengenes as the Centroids

Botía et al. [15] proposed k-means clustering as an additional processing step to the standard WGCNA. Their definition of k-means used eigengenes as centroids. These specific types of networks separate upregulated genes from downregulated genes in different modules. In this paper, for the purposes of comparison with the k-module algorithm, which is also an additional processing step tot he standard WGCNA, we use an algorithm that uses eigengenes as the centroids [15], but that is not limited to specific types of network. For convenience, we call it the k-eigengene algorithm. The detailed description of the steps of the k-eigengene algorithm is as follows: The modules obtained by WGCNA are the input of the k-eigengene algorithm. (1) Calculate the correlation coefficients between the genes and the eigengenes. (2) Assign the module label of each $g_{i}$ as:

(3) $$\arg\max_{j}\left|cor\left(g_{i},eg_{j}\right)\right|$$

where $eg_{j}$ is the eigengene in the *j*th module. (3) Then, repeat the previous two steps until none of the cluster assignments change or the set maximum number of iterations is reached.

### 2.4. Silhouette Coefficient and Dunn Index

Both k-eigengene and k-module algorithms are derived from the k-means algorithm, so the silhouette coefficient and the Dunn index are the common and effective internal measures for assessing the validity of clustering.

The silhouette value is a measure of the similarity of an observation to its own cluster compared to other clusters, and its definition can be found in [19]. The silhouette coefficient value ranges from −1 to 1. A value of 1 means the clusters are well apart from each other and are clearly distinguished, a value of 0 means the distance between clusters is not significant, and a value of −1 means clusters are assigned incorrectly. The Dunn Index is a metric for judging a clustering algorithm; it is the ratio of the smallest distance between observations in different clusters to the largest intra-cluster distance. A higher Dunn Index implies better clustering. The details of the Dunn Index can be found in [20,21].

### 2.5. Database for Annotation, Visualizationm and Integrated Discovery Enrichment Analysis

The Database for Annotation, Visualization and Integrated Discovery (DAVID; http://david.abcc.ncifcrf.gov/) is a database of biological information [22,23]. It integrates biological data and analytical tools, provides systematic and integrated biological function annotation information for large-scale gene and protein lists, and helps users extract biological information. Here, we used the enrichment score in the DAVID Functional Annotation Clustering Tool. The tool collects and integrates annotation terms from 14 public annotation categories (including gene ontology and Kyoto Encyclopedia of Genes and Genomes (KEGG) pathways). The functional annotation clustering report groups/displays similar annotations together, which clarifies the biology. The group enrichment score—the geometric mean (in-log scale) of the members’ *p*-values in a corresponding annotation cluster—is used to rank their biological significance.

### 2.6. Module Stability

To test the stability of the modules constructed by WGCNA and the k-module algorithm, we randomly divided the total dataset into two sets and each half was independently processed using both algorithms. Then, we observed the module preservation between each partition of the dataset for the different algorithms through the colour coding of −log(p) in the figures in Section 3.5, where *p* is the Fisher’s exact test *p*-value for the overlap between two modules. The Fisher’s exact test that was used to detect significant overlap between modules was the one-sided version. The depth of the red color represents the size of the *p*-value: the darker the red, the smaller the *p*-value and the more significant the overlap. Module stability was documented using R code [6] found at https://labs.genetics.ucla.edu/horvath/htdocs/CoexpressionNetwork/Rpackages/WGCNA/Tutorials/Consensus-RelateToFemMods.pdf.

The approach above, considering such as something like two-fold cross-validation, may be influenced by randomness. To reduce the influence of randomness, we presented a method, five-fold cross-validation, by further randomly splitting each dataset into k=5 folds and calculating the number of modules with preservation significance greater than 50 among the modules in all pair of sets.

### 2.7. Datasets

A total of six datasets were primarily used in this study. Of these, two were from microarray datasets and the other four were from RNA-Seq datasets. The two microarray datasets were collected from a large panel of mice, one from control and treated macrophages, and the other from livers. Genes involved in the regulation of inflammatory responses and gene–environment interactions were identified in macrophages from a set of mouse inbred strains, termed the hybrid mouse diversity panel (HMDP), while confounding factors such as environmental variation were minimized, making these datasets ideal for network biology and module identification. Macrophages were exposed to oxidized 1-palmitoyl-2-arachidonoyl-sn-glycero-3-phosphatidylcholine (OxPAPC) or control treatment conditions [24,25,26]. Here, we analyzed the control and OxPAPC treatment data (329 samples), which were normalized using the robust multi-array average (RMA) method. The liver dataset was taken from the contributors Bennett and Ghazalpour. Expression profiles were obtained from 99 strains of inbred and recombinant inbred mice. Most were assayed in triplicate [27,28]. The GPL8759 Affymetrix HT Mouse Genome 430A Array platform was used, and the data were normalized using the RMA method, comprising 288 samples in total. The microarray data and liver expression profile are available at http://www.ncbi.nlm.nih.gov/geo/ under accession numbers GSE38705 and GSE16780, respectively. Gene expression RNA-Seq of breast cancer and pancreatic cancer was performed using The Cancer Genome Atlas (TCGA) Research Network (https://www.cancer.gov/tcga). This dataset shows gene-level transcription estimates as $log_{2}\left(x+1\right)$ and transformed RNA-Seq by expectation-maximization (RSEM)-normalized counts. Another two gene expression RNA-Seqs of grape and *Arabidopsis* are available at https://atted.jp/. This dataset was transformed into RNA-Seq by Matataki-normalization.

Distance correlation is still a relatively expensive computation. One major limitation of the calculation of the distance correlation coefficient is its large computation time, $O(\frac{1}{2}n^{2}\times m^{2})$, where *n* is the number of genes and *m* is the number of samples. To calculate the distance correlation coefficient in a reasonable amount of time, it is important to limit the genes selected to the smallest informative set. For the six datasets, we calculated the coefficient of variation (CV) for the gene expression profiles and reduced our datasets to relevant genes by first selecting profiles with above-average intensity. Then, we reduced the microarray dataset to relevant genes by selecting probes with greater than 5% coefficients of variation, yielding 3611 genes for the macrophage dataset. Similarly, for the liver dataset, we selected 3089 genes for analysis. For the breast cancer dataset, we selected CV values greater than 10%, and finally obtained 3475 genes and 637 samples. For the pancreatic cancer dataset, the selection of probes with a CV greater than 10% resulted in 3029 genes and 183 samples. For the grape dataset, we selected CV values above 2.5% and finally obtained 3664 genes and 258 samples. For the *Arabidopsis* dataset, we sampled 500 samples at random and the selection of probes with a CV greater than 12% resulted in 2915 genes. The lists of the selected genes for all datasets are provided in Supplementary Material 2.

## 3. Results and Discussion

### 3.1. k-Module Algorithm Assigns the Gene to the Module with the Highest Mean Connectivity

In this paper, we considered three algorithms: the standard WGCNA, WGCNA with the k-eigengene, and WGCNA with the k-module as the additional processing step. For convenience, we simply denote them as WGCNA, k-eigengene, and k-module in the following text, respectively. For the six datasets, the selected soft thresholding power is shown in Supplementary Material 3 (Table S1). The goal of the k-module method is to construct clusters with highly inter-connected genes. The proportion of genes assigned to the module with the highest mean connectivity is shown in Supplementary Material 4 (Table S2). The k-module algorithm aims to assign all genes to the module with the highest mean connectivity. The goal of the k-module method is global, so all modules are involved in the measurement. Hierarchical clustering does not have a global goal. The goal of the k-eigengene algorithm is global; different from the k-module method, it assigns genes to the module with the highest correlation between gene and eigengene. So, for the WGCNA and k-eigengene algorithms, some genes were not assigned to the module with the highest mean connectivity.

**Figure 2:**
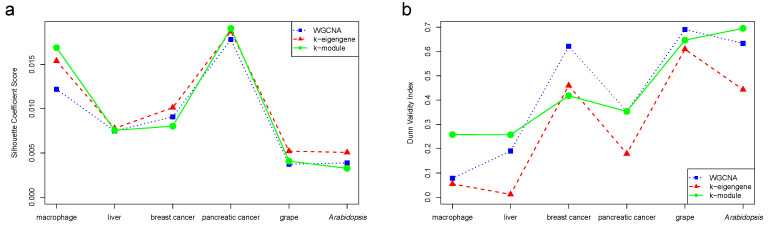
Silhouette coefficient score (**a**) and Dunn validity index (**b**) of WGCNA, k-eigengene, and k-module algorithms. The evaluation value obtained by the k-module algorithm was the highest in most of the datasets.

In this section, we simultaneously used the silhouette coefficient and the Dunn index to evaluate the quality of the WGCNA, k-eigengene, and k-module clustering results. As shown in Figure 2, the evaluation results were different for the six datasets. The clustering results of the k-module had the highest silhouette coefficient score in the macrophage and pancreatic cancer datasets, and the highest Dunn index in the macrophage, liver, pancreatic cancer, and *Arabidopsis* datasets. In other words, the k-module algorithm obtained the highest evaluation values in most of the datasets. For the breast cancer dataset, the WGCNA algorithm alone assigned 99.02% of the genes to the most highly connected module. The optimization effect of the k-module algorithm with WGCNA was lower, and fewer genes moved in the module. The k-module algorithm changed 2.42% of the gene labels. Although the evaluation score of the k-module algorithm was the lowest in the breast cancer dataset, the clustering results were similar to those of WGCNA.

### 3.2. Computational Complexity and Number of Iterations

To discuss the computational complexity, we divided WGCNA with the k-module as an additional processing step into three sequential observations: (1) calculating the similarity co-expression matrix with the distance correlation, the computational complexity is $O(\frac{1}{2}n^{2}\times m^{2})$, where *n* is the number of genes and *m* is the number of samples. (2) The computational complexity of standard WGCNA is $O(n^{2}$) [1,15]. (3) The computational complexity of k-module algorithms is O(n×k×ite), where *k* is the number of clusters (modules) and ite is the number of iterations.

The standard WGCNA method yielded different numbers of modules with different datasets. The number of modules and the gene count of each module are shown in Supplementary Material 5 (Table S3). Generally, the k-module and k-eigengene algorithms did not change the number of modules. For the k-module method, an extreme case where a small module may have all its genes reassigned to other modules is theoretically possible, but we did not observe this situation in any of our experiments. We designed a stopping criterion for the k-module algorithm based on the minimum number of misplaced genes being set to 0. However, we note that a situation may exist where the algorithm may fall into an infinite loop without reaching the desired state (i.e., changing the same genes from one module to another and back again) [15]. The k-module algorithm tries to reach the desired value for misplaced genes, but always within a limited number of iterations. We did not observe the mentioned infinite loop situation in any of our experiments with the k-module algorithm. For the k-eigengene algorithm, an infinite loop occurred when we used it to process half of the macrophage dataset in the Section 3.5. For the six datasets, the numbers of iterations of the two algorithms are shown in Table 1. Compared with the k-eigengene algorithm, the k-module algorithm had fewer iterations and the algorithm complexity was significantly lower for most of the datasets.

In principle, we used a PC (single-core CPU with i7 core) to calculate the distance correlation coefficients between the more than 3000 genes collected from approximately 300 samples. The process required approximately 9 h, which was acceptable. The standard WGCNA, k-eigengene, and k-module algorithms could thus obtain clustering results in a few minutes.

### 3.3. The k-Module Algorithm Readjusts a Small Number of Decisions

The dynamic tree cut method succeeded at identifying branches that could not have been identified using the static cut method. Prior studies provided indirect evidence that the resulting clusters are biologically meaningful [11]. The purpose of the k-module algorithm was to readjust the results of hierarchical clustering. Generally, since few decisions needed to be readjusted in hierarchical clustering, the number of gene labels corrected by the optimized algorithm will not be large. The change rate of gene labels obtained by the k-eigengene and k-module algorithms is shown in Table 2.

In Table 2, for the six datasets, the rates of change in gene labels by the k-eigengene algorithm were higher. Especially for the macrophage and liver datasets, the k-eigengene algorithm changed nearly half of all labels, whereas the rate of gene changes in the k-module algorithm was around 20%. Thus, the k-module algorithm readjusted a small number of decisions. For the breast cancer dataset, WGCNA assigned 99.02% of the genes into the module with the highest mean connectivity; thus, it was reasonable for the k-module algorithm to change only 2.42% of gene labels.

### 3.4. Gene Enrichment Comparison

**Figure 3:**
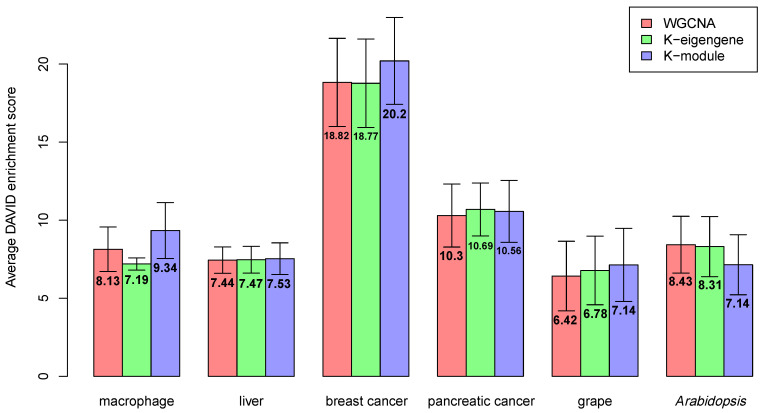
Average Database for Annotation, Visualization, and Integrated Discovery (DAVID) enrichment score of modules obtained by WGCNA, k-eigengene, and k-module algorithms. The enrichment score obtained by the k-module algorithm was the highest in most of the datasets.

Co-expressed genes tend to be involved in the same biological processes [29]. The network modules often have a biological interpretation in the sense that the modules are highly enriched in genes with a common functional annotation [30]. Ideal modules should be highly enriched for specific gene categories [18]. In this paper, we used DAVID [22,23] for enrichment analysis and took the enrichment score in the DAVID Functional Annotation Clustering Tool. The overall enrichment score for the module was based on the expression analysis systematic explorer (EASE) score of each term member: the higher the score, the more enriched the module. Next, we measured the enrichment of the co-expression network using the average DAVID enrichment score for all modules. As the enrichment score is an important evaluation of the modules’ rationality, we discuss the enrichment scores of the gene co-expression networks constructed by WGCNA, the k-eigengene, and the k-module algorithms. The average DAVID enrichment scores of modules obtained by WGCNA, the k-eigengene, and the k-module algorithms are shown in Figure 3. The gene number and DAVID enrichment score of each module can be found in Supplementary Material 5 (Table S3).

**Figure 4:**
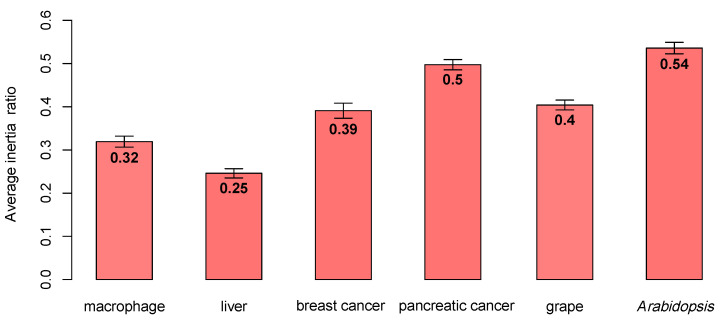
The average proportion of variance captured by eigengenes obtained by the k-eigengene algorithm. The proportion of variance in the pancreatic cancer and *Arabidopsis* datasets was the highest.

In Figure 3, for the macrophage, breast cancer, and grape datasets, the enrichment scores of the three algorithms were significantly different. The k-module algorithm had the highest enrichment score. For the liver dataset, the k-module algorithm also had the highest enrichment score, while the difference in the enrichment scores between these three methods was small. For the pancreatic cancer and *Arabidopsis* datasets, the enrichment score of the k-eigengene algorithm was higher than that of k-module, where the score of the k-eigengene algorithm was the highest in the pancreatic cancer dataset. If the eigengenes contained less module information, they would be unsuitable for use as centroids. Therefore, in the pancreatic cancer and *Arabidopsis* datasets, the eigengenes may contain more information about the module. To verify this conclusion, for each dataset, we counted the average proportion of variance captured by the eigengenes (the first principal component) in each module; the results are shown in Figure 4. As shown in Figure 4, the average proportion of variance captured by the eigengenes in the pancreatic cancer and *Arabidopsis* datasets was about 0.5, and for the other four datasets, its average proportion of variance was not more than 0.4. Therefore, the eigengenes contained more information about modules in the pancreatic cancer dataset, and this may be the reason for the higher enrichment score of the k-eigengene algorithm in the two datasets.

In most cases, the enrichment score of the modules obtained by the k-module algorithm was higher, so these modules had more significant biological significance and were more reasonable. When the average proportion of variance captured by the eigengenes was high, the k-eigengene algorithm was a useful alternative.

### 3.5. Stability Analysis

**Figure 5:**
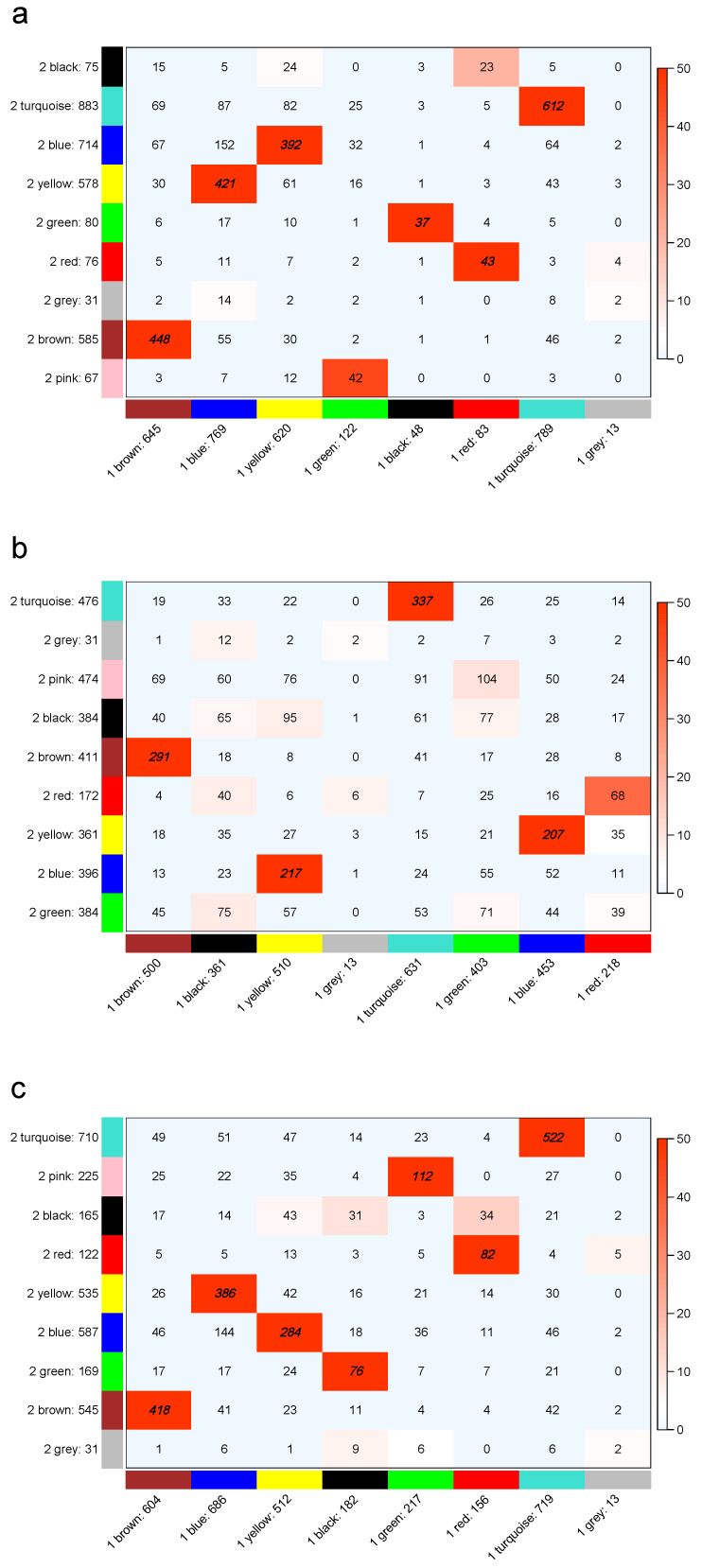
Module preservation between even partitioning of the liver dataset for WGCNA (**a**), k-eigengene (**b**) and k-module (**c**). All cells with a color depth below −log10(0.05) are shown as light blue, while the other cells maintain a color gradient from white to red. The modules with preservation significance greater than 50 have the numbers printed in bold and italic. The k-module algorithm has reasonable module preservation statistics.

To determine the stability of the modules obtained by WGCNA, the k-eigengene, and the k-module algorithms, we randomly divided the liver data into two parts, and each half was independently processed using the three algorithms. To observe the module preservation between each partition of the liver dataset for the three algorithms, we examined the preservation significance, as shown in Figure 5. The numbers in Figure 5 indicate gene counts in the interaction of the corresponding modules. As the grey module consisted of genes not assigned to any module, it did not overlap with other modules.

WGCNA yielded different numbers of modules (eight vs. nine) for the two halves of the liver dataset. The k-eigengene and k-module algorithms were the optimization of WGCNA, and the numbers of modules were the same as in WGCNA. There were six modules with preservation significance greater than 50 in WGCNA (Figure 5a), four modules in the k-eigengene algorithm (Figure 5b), and seven modules in the k-module algorithm (Figure 5c). For the liver dataset, the k-module algorithm was more stable than the WGCNA and k-eigengene algorithms in terms of preservation significance.

We counted the number of modules with preservation significance greater than 50 among the modules in five other datasets, and the stability analysis results are provided in Supplementary Material 6 (Figure S1). The k-module algorithm was also the most stable in the macrophage and grape datasets. In the breast cancer dataset, the three algorithms had the same stability due to WGCNA assigning most of the genes into the module with the highest mean connectivity; the optimization algorithms only changed a few gene labels. In the pancreatic cancer dataset, the WGCNA and k-module algorithms had the same stability and they were more stable than the k-eigengene algorithm. In the *Arabidopsis* dataset, the k-eigengene algorithm was significantly the most stable.

The data of the two halves were different in each test because of the randomness of the division. Thus, variation in the modules in each test led to slight differences between each stability analysis result. To reduce the influence of randomness, we used five-fold cross-validation, and the average values and ranges of the numbers of modules with preservation significance greater than 50 among the modules in each of the two sets are shown in Table 3. Since there were too few samples in the pancreatic cancer dataset for five-fold cross-validation, sometimes SFT could not be achieved. So, we simply maintained their two-fold split, but generated 10 random (but distinct) two-way splits. As shown in Table 3, decreasing the samples may lead to the reduction in stability. For example, the numbers of modules with high preservation significance in the liver dataset decreased to 3.6, 3.2, and 3.9 from 6, 4, and 7 for WGCNA, k-eigengene, and k-module methods, respectively. In the macrophage, liver, grape, and pancreatic cancer datasets, the k-module method had a greater value than WGCNA and k-eigengene methods. In the *Arabidopsis* dataset, the k-eigengene method had a prominently highest value. From Figure 4, the average proportion of variance captured by eigengenes obtained by the k-eigengene algorithm in the *Arabidopsis* dataset was the highest. Therefore, the high average proportion of variance may lead to high stability.

### 3.6. Corresponding Results Based on Pearson Correlation Coefficients

Since the distance correlation coefficients work for both linear and nonlinear dependencies between two vectors, the correlation between the gene expression profiles was calculated using the distance correlation in this study. However, Pearson correlation coefficients are popular in computing the similarity matrix. Therefore, we provide the corresponding Pearson correlation coefficient results in Supplementary Material 7.

From the figures and tables, the different correlation coefficients indicate the differences in the clustering results, but the k-module and k-eigengene algorithms perform very similarly regardless of the correlation coefficient used. When the Pearson coefficient was used, the silhouette coefficient value of the k-module algorithm was not the highest in most cases, but when compared in terms of the Dunn index and enrichment analysis, the k-module algorithm performed better; these results are similar to those of distance correlation. When compared in terms of module stability, k-eigengene performed similar to the k-module algorithm when the Pearson correlation coefficient was used. The average proportion of variance captured by the eigengenes obtained by the k-eigengene algorithm in the *Arabidopsis* dataset was the highest; the stability of the k-eigengene algorithm was significantly better than that of the other two methods.

## 4. Conclusions

In this paper, we presented a new approach to improve the results of standard WGCNA. This approach provides two major improvements upon previous works. The first is the use of distance correlation to calculate the correlations between the gene expression profiles. The second is the novel k-module algorithm, which optimizes the clustering modules obtained by WGCNA.

The validity of the algorithm was verified using microarray and RNA-Seq data. The k-module algorithm re-adjusts the results of hierarchical clustering and retains the advantages of the dynamic tree cut method. The number of modules obtained by the dynamic tree cut method did not change, and only a small number of module labels of genes changed. The k-module algorithm can assign all genes to the module in which a gene has a high mean connectivity to the other genes. It has fewer iterations, which results in lower complexity. Finally, we verified that the modules obtained by the k-module algorithm had higher enrichment scores and strong stability.

Compared with the k-eigengene algorithm, the k-module algorithm performs well without limiting the specific types of networks, separating up and downregulated genes into different modules. The performance of the two optimization algorithms is different on different datasets. For the macrophage and liver datasets, the average proportion of variance captured by the eigengenes was lower; the performance of the k-module method with respect to enrichment and stability analysis was the best. For the pancreatic cancer dataset, the average enrichment score of the k-eigengene algorithm was the highest. For the *Arabidopsis* dataset, the k-eigengene method had prominently higher stability. The pancreatic cancer and *Arabidopsis* datasets had a high average proportion of variance captured by the eigengenes. In practice, we suggest that the k-eigengene algorithm should be selected when the average proportion of variance captured by the eigengene in each module is high (about 50%), and the k-module could be applied to other situations.

The k-module algorithm improves the computational strategy and expands the general applicability of WCGNA. As an improvement in hierarchical clustering, the method can be applied not only to gene co-expression networks, but also to any other general clustering algorithms based on similarity matrices or network generation.

## Figures and Tables

**Table 1:** The numbers of iterations of the k-eigengene and k-module algorithms. The k-module algorithm has fewer iterations in most of the datasets.

Dataset	k-Eigengene	k-Module
macrophage	33	18
liver	59	15
breast cancer	8	9
pancreatic cancer	17	11
grape	24	13
*Arabidopsis*	42	14

**Table 2:** The change rate of gene labels obtained by the k-eigengene and k-module algorithms. The k-module changes a small number of the gene labels.

Dataset	k-Eigengene	k-Module
macrophage	45.58%	23.73%
liver	43.44%	17.19%
breast cancer	15.74%	2.42%
pancreatic cancer	27.73%	13.73%
grape	36.68%	7.80%
*Arabidopsis*	34.74%	26.38%

**Table 3:** The average values and ranges of the numbers of modules with preservation significance greater than 50 among the modules in each of the two sets.

Dataset	WGCNA	k-Eigengene	k-Module
macrophage	5.0 (4–6)	5.7 (4–7)	5.8 (5–7)
liver	3.6 (3–4)	3.2 (3–4)	3.9 (3–5)
breast cancer	7.7 (6–9)	8.5 (7–10)	8.4 (7–9)
pancreatic cancer	10.1 (9–11)	10.2 (7–12)	10.6 (9–11)
grape	5.1 (3–7)	5.8 (3–10)	6.0 (3–8)
*Arabidopsis*	9.5 (8–13)	11.9 (8–15)	9.4 (7–12)

## Data Availability

Data available in publicly accessible repositories. The microarray data and liver expression profile are available at http://www.ncbi.nlm.nih.gov/geo/ under accession numbers GSE38705 and GSE16780; The breast cancer and pancreatic cancer was performed using The Cancer Genome Atlas (TCGA) Research Network at https://www.cancer.gov/tcga; The grape and *Arabidopsis* are available at https://atted.jp/.

## Supplementary Materials

The following are available online at https://www.mdpi.com/2073-4425/12/1/87/s1, R Code: R Code of the k-module algorithm. Gene ID: The gene ID of six datasets. Table S1: Soft thresholding power. Table S2: The proportion of genes assigned to the module with the highest mean connectivity. Table S3: The gene number and DAVID enrichment score in each module. Figure S1: Module preservation between even partitioning of each datasets. Results based on Pearson: Tables and figures based on Pearson coefficient.

## Abbreviations

The following abbreviations are used in this manuscript:

WGCNA	Weighted gene co-expression network analysis
DAVID	Database for Annotation, Visualization, and Integrated Discovery
SFT	scale-free topology
KEGG	Kyoto Encyclopedia of Genes and Genomes
HMDP	Hybrid mouse diversity panel
OxPAPC	Oxidized 1-palmitoyl-2-arachidonoyl-sn-phosphatidylcholine
RMA	Robust multi-array average
TCGA	The Cancer Genome Atlas
RSEM	RNA-Seq by expectation-maximization
CV	Coefficient of variation
EASE	Expression analysis systematic explorer
