# Supplementary material for: K-Module Algorithm: An Additional Step to Improve the Clustering Results of WGCNA Co-Expression Networks
Source: Genes (Basel). 2021 Jan 12;12(1):87. doi: 10.3390/genes12010087 (PMC7828115; doi:10.3390/genes12010087)
Supplement: Supplementary file 1 [file genes-12-00087-s001.zip › Supplementary File/Supplementary Material 1ú║R Code of the k-Module Algorithm.docx]

R Code of the k-Module Algorithm

# K-module function

# author: Hou Jie; # date: 2020-7-17

# College of Intelligent Systems Science and Engineering, Harbin Engineering University

# input: dynamicMods, adjacency (The result obtained by WGCNA), and MaxIterations

# output: dynamicColors (Modules Colors optimized by k-module algorithm)

# example: First run the WGCNA algorithm to get the cluster(module) label and adjacency matrix, then run the following code

# source("Kmodule.R")

# dynamicColors<-Kmodule(dynamicMods, adjacency, 100)

Kmodule<-function(dynamicMods, adjacency, MaxIterations){

nIteration=0 # iterations

continue.change=TRUE

cluster.vector<-dynamicMods# Save the cluster(module) label for each gene

initialClusterColors=lastClusterColors=clusterColors = labels2colors(cluster.vector)# Save the cluster(module) Color for each gene

colorlevels=unique(clusterColors)

nCluster<-length(colorlevels) # The number of module

nGenes<-length(clusterColors) # Number of genes

error.matrix<-matrix(0,nrow=nGenes,ncol=nCluster) # Record the average connectivity of each gene to each cluster(module)

colnames(error.matrix)<-colorlevels

while(continue.change){

nChange=0

nLastChange=0

# genes correlation for each module

for (i in c(1:nCluster))

{

whichmodule=colorlevels[[i]];

restrict1 = (clusterColors==whichmodule);

Alldegrees1=intramodularConnectivity(adjacency, clusterColors)#calculating the intramodular connectivity for each gene

nmodule<-length(which(restrict1==TRUE))

moduleLabel<-as.numeric(rownames(Alldegrees1)[restrict1])

error.matrix[,i]<-rowSums(adjacency[,moduleLabel])/nmodule

}

error.matrix[, which(colorlevels=="grey")]=0 # The gene in gray module remains the same

#Redefine the cluster(module) to which each gene belongs

for (i in 1:nGenes){

if(cluster.vector[i]!=0){

clusterColors[i]<-colorlevels[which.max(error.matrix[i, ])]

}

if(clusterColors[i] != initialClusterColors[i]){nChange<-nChange+1}

if(clusterColors[i] != lastClusterColors[i]){nLastChange<-nLastChange+1}

}

lastClusterColors<-clusterColors

nIteration=nIteration+1

continue.change=all(nIteration<MaxIterations & nLastChange>0)

}

dynamicColors<-clusterColors

return(dynamicColors)

}
