# Supplementary material for: K-Module Algorithm: An Additional Step to Improve the Clustering Results of WGCNA Co-Expression Networks
Source: Genes (Basel). 2021 Jan 12;12(1):87. doi: 10.3390/genes12010087 (PMC7828115; doi:10.3390/genes12010087)
Supplement: Supplementary file 1 [file genes-12-00087-s001.zip › Supplementary File/Supplementary Material 6ú║FigureS1.pdf]

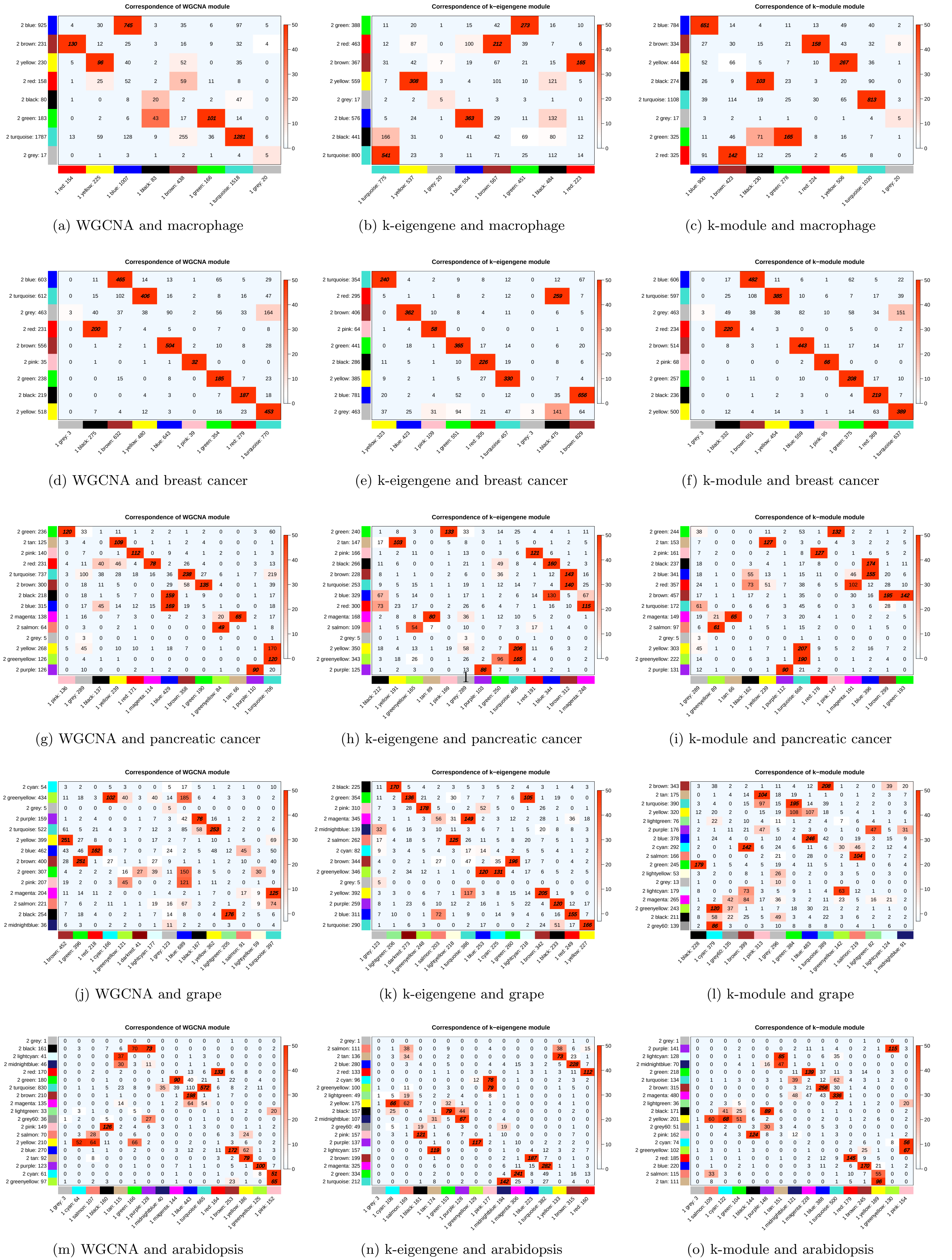

(a) WGCNA and macrophage (b) k-eigengene and macrophage (c) k-module and macrophage

(d) WGCNA and breast cancer (e) k-eigengene and breast cancer (f) k-module and breast cancer

(g) WGCNA and pancreatic cancer (h) k-eigengene and pancreatic cancer (i) k-module and pancreatic cancer

(j) WGCNA and grape (k) k-eigengene and grape (l) k-module and grape

(m) WGCNA and arabidopsis (n) k-eigengene and arabidopsis (o) k-module and arabidopsis

Figure s1: Module preservation between even partitioning of each datasets. The number of modules with preservation signicance greater than 50 obtained by WGCNA, k-eigengene and k-module algorithms is: 5 vs 6 vs 7 in macrophage, 8 vs 8 vs 8 in breast cancer, 12 vs 11 vs 12 in pancreatic cancer, 10 vs 10 vs 11 in grape, 11 vs 15 vs 13 in arabidopsis.
