# Supplementary material for: K-Module Algorithm: An Additional Step to Improve the Clustering Results of WGCNA Co-Expression Networks
Source: Genes (Basel). 2021 Jan 12;12(1):87. doi: 10.3390/genes12010087 (PMC7828115; doi:10.3390/genes12010087)
Supplement: Supplementary file 1 [file genes-12-00087-s001.zip › Supplementary File/Supplementary Material 7ú║ Results based on Pearson.pdf]

Supplementary Material 7 : Results based on Pearson

The correlation between the gene expression profiles was calculated utilizing the distance correlation in this paper. Nevertheless, Pearson correlation coefficients are popular in computing the similarity matrix. Therefore, we provided the corresponding results obtained by Pearson coefficient as follows, which can be compared with the results based on distance correlation. The different correlation coefficients lead to the difference of clustering results. When Pearson coefficient was used, the silhouette coefficient value of the k-module algorithm is not the highest in most cases, but in other evaluation methods, such as Dunn index, enrichment analysis and stability analysis, the results based on Pearson are similar to the ones of distance correlation.

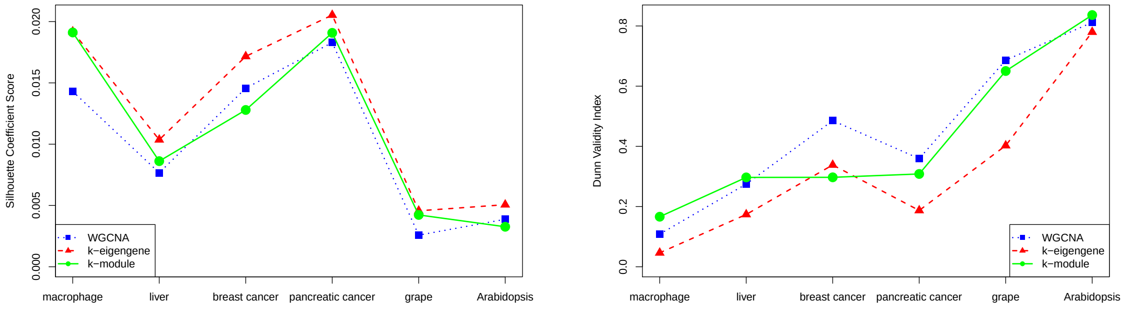

(a) Silhouette coefficient score

(b) Dunn validity index

Figure 2. Silhouette coefficient and Dunn index of WGCNA, k-eigengene, and k-module algorithm.

Table 1. The numbers of iterations of the k-eigengene and k-module algorithms. The k-module algorithm has fewer iterations in most of the datasets.

**Dataset**	**k-eigengene**	**k-module**
macrophage	29	6
liver	75	6
breast cancer	8	9
pancreatic cancer	17	12
grape	22	13
arabidopsis	25	5

Table 2. The change rate of gene labels obtained by the k-eigengene algorithm and k-module algorithm. The k-module changes a small number of the gene labels.

**Dataset**	**k-eigengene**	**k-module**
macrophage	34.25%	16.31%
liver	58.08%	19.88%
breast cancer	21.18%	4.78%
pancreatic cancer	23.04%	7.23%
grape	29.09%	9.25%
arabidopsis	31.59%	1.13%

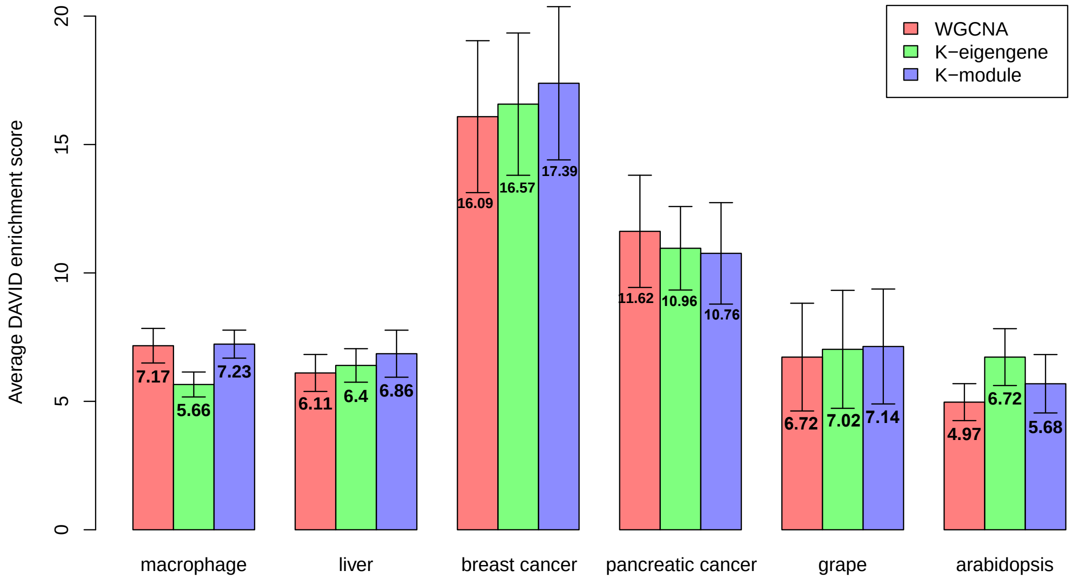

Figure 3. Average Database for Annotation, Visualization and Integrated Discovery (DAVID) enrichment score of modules obtained by WGCNA, k-eigengene, and k-module algorithm. The enrichment score obtained by the k-module algorithm was the highest in most of the datasets.

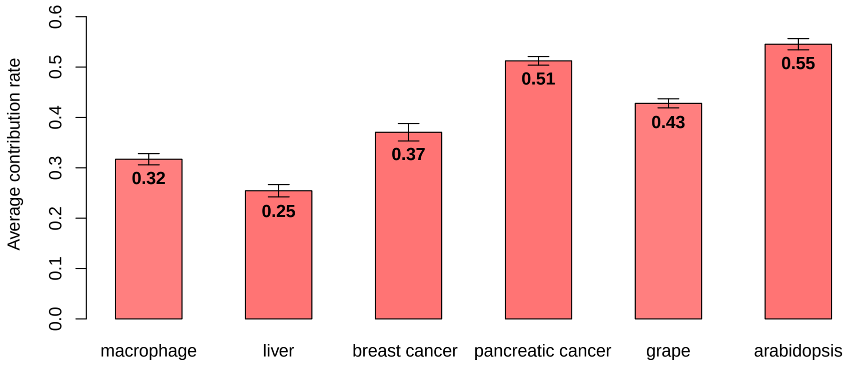

Figure 4. The average contribution ratio of eigengenes obtained by the k-eigengene algorithm. The ratio in the pancreatic cancer and arabidopsis dataset was the highest.

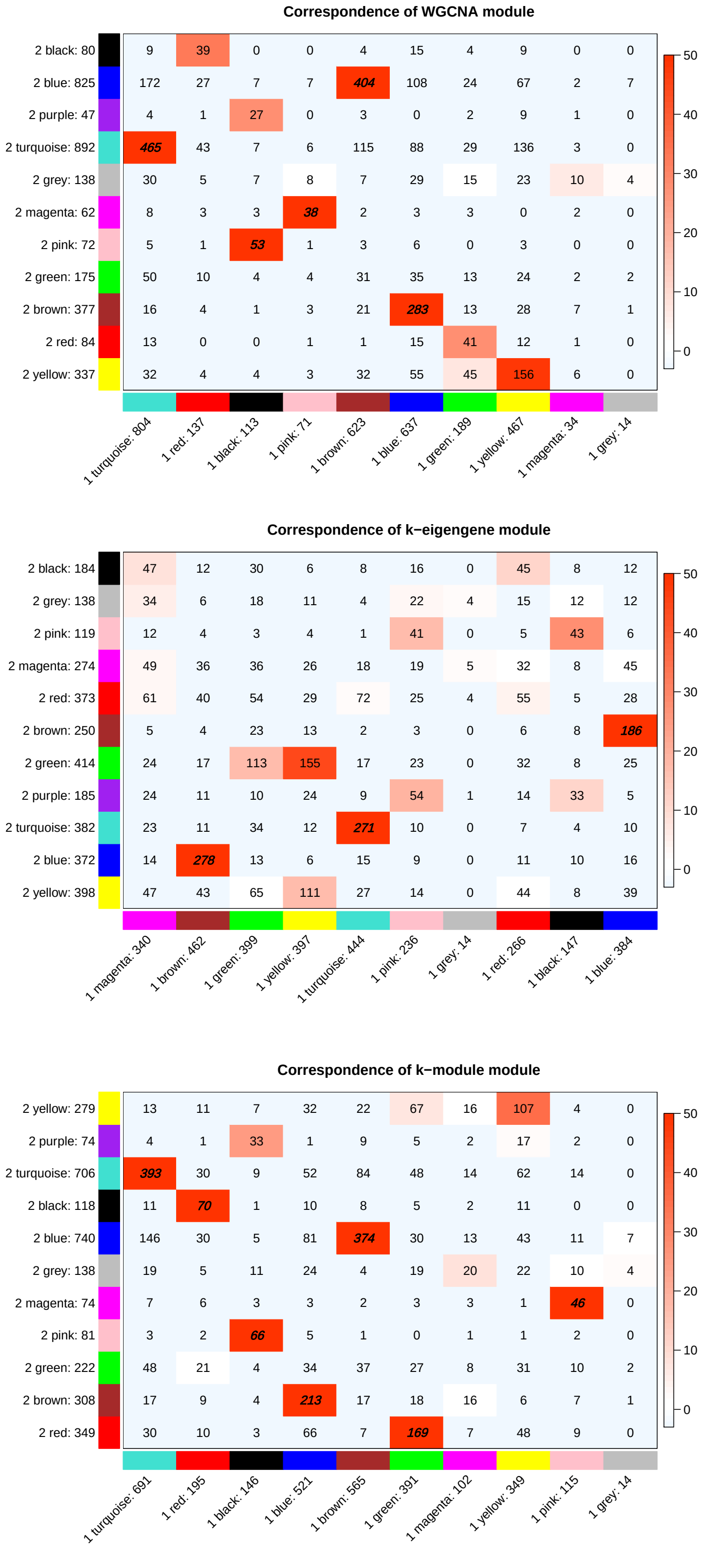

Figure 5. Module preservation between even partitioning of the liver dataset, showing that the k-module has reasonable module preservation statistics.

Table 3. The average value of the numbers of modules with preservation significance greater than 50 among the modules in per two sets. In liver, pancreatic cancer and grape datasets, the values of k-module method are greater than WGCNA and k-eigengene method; In macrophage, breast cancer and arabidopsis, k-eigengene method has the prominent highest value.

Dataset	WGCNA	k-eigengene	k-module
macrophage	4.5 (3-7)	6.4 (5-8)	5.1 (3-7)
liver	3.2 (2-4)	3.1 (2-4)	3.3 (2-5)
breast cancer	7.4 (6-8)	8.4 (7-9)	7.4 (7-8)
pancreatic cancer	10.7 (8-13)	10 (8-14)	10.9 (9-14)
grape	5.1 (3-8)	5.1 (3-7)	5.5 (3-8)
arabidopsis	9.8 (7-13)	12.5 (11-14)	10.1 (8-13)
